# Symmetric Diffeomorphic Modeling of Longitudinal Structural MRI

**DOI:** 10.3389/fnins.2012.00197

**Published:** 2013-02-05

**Authors:** John Ashburner, Gerard R. Ridgway

**Affiliations:** ^1^Wellcome Trust Centre for Neuroimaging, UCL Institute of NeurologyLondon, UK

**Keywords:** diffeomorphisms, geodesic shooting, shape modeling, non-linear registration, longitudinal registration, inverse consistency, symmetry, transitivity

## Abstract

This technology report describes the longitudinal registration approach that we intend to incorporate into SPM12. It essentially describes a group-wise intra-subject modeling framework, which combines diffeomorphic and rigid-body registration, incorporating a correction for the intensity inhomogeneity artifact usually seen in MRI data. Emphasis is placed on achieving internal consistency and accounting for many of the mathematical subtleties that most implementations overlook. The implementation was evaluated using examples from the *OASIS Longitudinal MRI Data in Non-demented and Demented Older Adults*.

## 1 Introduction

Growth, plasticity, aging, and degeneration are inherently longitudinal processes; while much can be learned by studying a cross-sectional sample of subjects at different stages of such processes, longitudinal data have well-established advantages in terms of increasing power and reducing confounds. To give just one example, cross-sectional studies of aging are challenged by the relative subtlety of changes over time compared to the large inter-individual variation, and they can never fully separate true aging effects from confounding effects of birth-year such as changes in nutrition. Longitudinal anatomical MRI provides a framework for characterizing many of the macroscopic brain changes in natural development and in response to disease or injury. A *MEDLINE* search for keywords “longitudinal,” “brain,” and “MRI” in the topic, gives over 1,500 hits.

Longitudinal data require appropriate statistical models, due to the dependence of repeated measurements within-subject. Beyond this, it is also recognized that there can be substantial gains in power from using image processing and modeling algorithms that are specifically designed for longitudinal data. For example, the boundary shift integral (Freeborough and Fox, 1997; Leung et al., 2012), which provides a direct measure of the difference between two brain volumes, has proven to be substantially more powerful than simple subtraction of the two brain segmentations upon which it is based. However, the reduction in measurement variability comes at a price of increased risk of various forms of bias. In particular, any technique that is not symmetric^1^ with respect to the multiple time-points has the potential to introduce false positive differences.

Concerns about asymmetry in pairwise image registration, related to one image being chosen as a (fixed) reference and the other as a (moving) source, first arose at the turn of the century (Ashburner et al., 1999; Cachier and Rey, 2000; Christensen and Johnson, 2001). In particular, Smith et al. (2001) discuss the potential use of the matrix square root of an affine transformation to derive a “half-way” space between two images^2^. This concept has since been generalized to more than two images, as described further below. Despite this early recognition of the potential problem, many (probably most) studies employing image registration for longitudinal data over the past decade have used methods with some form of asymmetry [usually registering the later time-point(s) to the baseline].

More recently, it has been empirically demonstrated that these theoretical concerns can indeed cause practical problems. Pairwise registration results have been shown to differ depending on which image is chosen as the reference (Thomas et al., 2009; Yushkevich et al., 2010). Results over three time-points appear to exhibit an additive bias, such that the majority of change occurs over the first interval, in a setting where such deceleration is not biologically plausible (Hua et al., 2011; Thompson et al., 2011). Intransitivity (where, for example, the sum of changes from A to B and B to C differs from the change estimated directly from A to C) can also be demonstrated (Leung et al., 2012). The increasingly significant potential of longitudinal MRI for clinical diagnosis, as a biomarker of disease progression and as an outcome measure in treatment trials, has led to an increased focus on this issue (Fox et al., 2011; Reuter and Fischl, 2011), along with on-going controversy (Holland et al., 2012).

Many of the solutions proposed for avoiding bias from asymmetry are restricted to two time-points, such as the formulation of Tagare et al. (2009). There are three distinct approaches for attempting to address asymmetry and intransitivity across more than two time-points: all time-points of all subjects can be treated independently, i.e., processed cross-sectionally (e.g., Giedd et al., 1999); each time-point can be registered to every other time-point within-subject, with appropriate adjustments to ensure consistency (e.g., Leung et al., 2012); or all time-points can be registered to some form of within-subject average image (e.g., Skrinjar et al., 2008; Reuter et al., 2012a). The first of these results in low or zero bias but very high variance, while the second is computationally infeasible for high-dimensional diffeomorphic image registration. We therefore propose a solution based on the third approach. Registration to an average is defined as an “indirect” approach by Tagare et al. (2009), in contrast to “direct” approaches which symmetrize an individual pairwise registration; however, in this work, we attempt to address some of the more subtle differential geometric issues raised by Tagare et al. (2009) in their direct setting. A further novelty of our approach is the unification of rigid registration, diffeomorphic registration, and correction of (differential) intensity inhomogeneity in a single generative model (in the same spirit as Ashburner and Friston, 2005). The algorithms described in this Technology Report will be made available in the Statistical Parametric Mapping (SPM)^3^ software with the next major release, SPM12.

## 2 Methods

Our proposed model involves combining rigid-body registration, intensity inhomogeneity correction, and non-linear diffeomorphic registration. Because of all the conditional dependencies among model parameters, naive approaches involving pipelines of steps are likely to lead to less optimal solutions. For example, accurate inhomogeneity correction requires the images to be aligned, whereas more accurate alignment is only possible after inhomogeneity correction. Our solution involves performing all steps in an interleaved fashion, such that model parameters are optimized together. Rigid-body and diffeomorphic registration are combined because the boundary conditions of the diffeomorphic registration do not permit rotations of the whole image. In addition, the inclusion of translations in the rigid-body model allows the diffeomorphic model to not penalize pure displacements.

In the remainder of this section, the individual components will be introduced separately, before proceeding to describe the combined model and some of the implementational details. First though, we say something about how the within-subject template space is defined.

### 2.1 Template position and dimensions

The SPM software uses the concept of voxel-to-world mappings. These are encoded in the image headers, and are used to determine the real world locations (**y**) of voxel indices (**x**). Usually, they are read from the “sform” fields of the NIfTI header^4^, and encode affine transformation matrices (**M**), such that

(1) $$\left[\begin{array}{c} y_{1}\\ y_{2}\\ y_{3}\\ 1 \end{array}\right]=\left[\begin{array}{cccc} m_{11} & m_{12} & m_{13} & m_{14}\\ m_{21} & m_{22} & m_{23} & m_{24}\\ m_{31} & m_{32} & m_{33} & m_{34}\\ 0 & 0 & 0 & 1 \end{array}\right]=\left[\begin{array}{c} x_{1}\\ x_{2}\\ x_{3}\\ 1 \end{array}\right]\cdot$$

Each of the individual images is assumed to have such a voxel-to-world mapping associated with it (**M***_n_*). The first step of the algorithm involves computing suitable dimensions and a voxel-to-world mapping (**M***_μ_*) for the within-subject template. The aim is to have this template in some form of average position such that bias introduced by interpolation is minimized.

As mentioned above, some pairwise registration approaches have suggested using square roots of transformation matrices for determining the half-way position for approximately symmetrizing the registration (Smith et al., 2001; Thomas et al., 2009; Yushkevich et al., 2010). Several forms of transformation are members of matrix Lie groups (Woods, 2003), so it is illustrative to consider one of the simplest Lie groups – the positive scalars under multiplication. As emphasized in Leung et al. (2012), the conventional square root of a positive scalar can be seen as the geometric mean of the scalar and unity (the identity for positive scalars). The simple geometric mean of a set of points can be shown to minimize the sum squared distances from the mean to all points under a logarithmic distance metric; this property defines a Fréchet mean, sometimes known as a Karcher mean, where the latter is only required to be at a local minimum (Woods, 2003). The geometric mean is also an exponential barycenter (Pennec and Arsigny, 2012), in the sense that the signed logarithmic discrepancies from the mean to all other points sum to zero. Considering now the Lie group of three-dimensional rotation matrices [known as SO(3), the special orthogonal group], an appropriate Riemannian distance metric and Fréchet mean (also the exponential barycenter) can be analogously defined (Moakher, 2002). Unfortunately, in the more general groups of rigid-body or affine transformations, it can be shown that no bi-invariant Riemannian metric exists^5^.

Three different compromises have been used in the literature to circumvent the lack of bi-invariant metric: Reuter et al. (2012b) compute a simple Euclidean mean of rigid-body transformations before using the singular value decomposition to factor out any resultant zooms and shears; (Leung et al., 2012) use Arsigny’s log-Euclidean mean (Arsigny, 2006), which is a distance-minimizing Fréchet mean under a sub-optimal metric that is not bi-invariant; (Woods, 2003) use the exponential barycenter (Pennec and Arsigny, 2012) which corresponds to a bi-invariant mean, relaxing the manifold to a semi-Riemannian one without a true metric (and hence without a Fréchet mean), but still having a set of tangent space vectors from the mean that sum to zero.

Our implementation is also based on the exponential barycenter, which in theory could be directly used to define the average position template. Unfortunately, because voxel sizes are not necessarily isotropic, there are some potential difficulties with using this. In particular, it is possible for the exponential barycenter to be a transform that can not be encoded by axis-aligned scaling (to account for voxel sizes), rotating, and translating (i.e., nine parameters). It would not be possible to encode such a matrix in the “qform” fields of a NIfTI header, and it would also make some of the subsequent processing slightly more involved. Therefore, the closest nine-parameter affine transform to the exponential barycenter is estimated via a Gauss-Newton optimization strategy that minimizes the Euclidean distance between the matrices.

Finally, the dimensions of the template are determined such that it easily covers the field of view of the images. This is achieved by projecting the locations of the corners of the images into the template space and finding the maximum and minimum values. Corresponding adjustments are also needed for **M***_μ_*, which involves changing the translations.

Once the dimensions, etc., of the template are defined, the algorithm can proceed to fit the combined model. The next three subsections outline the individual components, and how they would be optimized in isolation. This will be followed by a subsection about combining the components together into the unified model.

### 2.2 Group-wise rigid-body registration

Each image is considered as a scalar function of space, such that $f_{n}:\Omega_{f_{n}}\to \mathbb{R}$, where $\Omega_{f_{n}}\subseteq {\mathbb{R}}^{3}$. A series of *N* images ${\left\{f_{n}\right\}}_{n=1}^{N}$ may be modeled as a common template mean $\left(\mu :\Omega_{\mu }\to \mathbb{R}\right)$, which is rotated and translated by a rigid-body transformation. Additive Gaussian noise (of variance 1/λ*_n_*) is assumed to be constant over each image and known, but may vary from image to image.

When dealing with rigid-body transforms, it is useful to consider them in terms of their membership of the special Euclidean group in three dimensions [*SE*(3)]. Within this framework, a transformation matrix (**R_q_**) is constructed via an exponential mapping of the six parameters (**q**) that constitute the Lie algebra of the group.

(2) $$R_{\text{q}}=\exp\left[\begin{array}{cccc} 0 & q_{4} & -q_{5} & q_{1}\\ -q_{4} & 0 & q_{6} & q_{2}\\ q_{5} & -q_{6} & 0 & q_{3}\\ 0 & 0 & 0 & 0 \end{array}\right]$$

This involves a matrix exponential, rather than computing the exponential of each element. Although there are many ways to compute this (Moler and Van Loan, 2003), it is usually defined as

(3) $$\exp\text{Q}=\overset{\infty }{\underset{n=0}{\sum }}\frac{1}{n!}{\text{Q}}^{n}.$$

Rigid alignment among a set of images can be performed by estimating the optimal mapping between the template and each of the images. A mapping from voxel indices in the template, to those in the *n*th image, is given by the following affine transform:

(4) $$\xi_{q_{\text{n}}}\left(\text{x}\right)={\text{I}}_{3,4}{\text{M}}_{n}^{-1}{\text{R}}_{{\text{q}}_{n}}{\text{M}}_{\mu }\left[\begin{array}{c} \text{x}\\ \text{1} \end{array}\right],\text{where }{\text{I}}_{3,4}=\left[\begin{array}{ccc} 1 & 0 & 0\\ 0 & 1 & 0\\ 0 & 0 & 1 \end{array}\right].$$

This leads to the following objective function for group-wise rigid-body alignment:

(5) $$\varepsilon =\overset{N}{\underset{n=1}{\sum }}\frac{\lambda_{n}}{2}{∥f_{n}-\mu \left(\xi_{{\text{q}}_{n}}^{-1}\right)∥}^{2}.$$

Without constraints, this simple approach may lead to a variety of equivalent solutions in which the template is rotated and translated by some arbitrary amount. The Lie algebra of the *SE*(3) group is used to parameterize the transforms to make is easier factor out the Fréchet mean of all the rigid-body transforms, thus ensuring that the template remains in the average position. All that is required to achieve this is to ensure that $\sum_{n=1}^{N}{\text{q}}_{n}=0$, which can be achieved simply by subtracting the mean after all the **q***_n_* have been re-estimated.

For reasons that should become apparent later, we formulate the objective function as

(6) $$\begin{array}{rcll} \varepsilon & =\overset{N}{\underset{n=1}{\sum }}\frac{\lambda_{n}}{2}\left|\text{D}\xi_{{\text{q}}_{n}}\right|\int_{\text{x}\in \Omega_{n}^{\prime }}\;{\left(f_{n}\left(\xi_{{\text{q}}_{n}}\left(\text{x}\right)\right)-\mu \left(\text{x}\right)\right)}^{2}d\text{x}, & & \\ & \;\text{such}\;\text{that}\;\overset{N}{\underset{n=1}{\sum }}{\text{q}}_{n}=0. & \end{array}$$

In the above equation, the **D** operator refers to computing the Jacobian, the determinants of which are included to account for a change of variables. The field of view common to both the *n*th image and the template is denoted by $\Omega_{n}^{\prime }=\xi_{{\text{q}}_{n}}^{-1}\left(\Omega_{f_{n}}\right)\cap \Omega_{\mu }.$

The optimization strategy involves alternating between re-estimating the mean (*μ*), and then sequentially using this to re-estimate the registration parameters. The update of *μ* is achieved by differentiating equation (6) with respect to *μ* and solving.

(7) $$\begin{array}{rcll} \mu \left(\text{x}\right) & =\frac{\sum_{n=1}^{N}\;w_{n}\left(\text{x}\right)f_{n}\left(\xi_{q_{n}}\left(\text{x}\right)\right)}{\sum_{n=1}^{N}\;w_{n}\left(\text{x}\right)},\;\text{where} & & \\ & \;w_{n}\left(\text{x}\right)=\left\{\begin{array}{cc} \lambda_{n}\left|\text{D}\xi_{{\text{q}}_{n}}\right|\; & \text{if}\;\text{x}\in \Omega_{n}^{\prime }\\ 0\; & \text{otherwise}\\ \; \end{array}\right.. & \end{array}$$

A Gauss-Newton optimization iteration is then done for each of the *N* scans. Dropping the *n* subscripts for notational simplicity, this involves the following:

(8) $$\text{q}\leftarrow \text{q}-{\left({\frac{\partial^{2}\varepsilon }{\partial {\text{q}}^{2}}|}_{\text{q}}\right)}^{-1}\left({\frac{\partial \varepsilon }{\partial \text{q}}|}_{\text{q}}\right).$$

It requires the vector of first derivatives, which are computed by differentiating equation (5) with respect to **q**, and applying the same change of variables as earlier (see Appendix A), giving:

(9) $$\frac{\partial \varepsilon }{\partial q_{i}}=\int_{\text{x}\in \Omega^{\prime }}\;a\left(\text{x}\right)\text{g}\left(\text{x}\right)\cdot {\text{h}}_{i}\left(\text{x}\right)d\text{x}$$

where *a* = λ|**D**ξ**_q_**|(*f*(ξ**_q_**) − *μ*), **g** = ▽*μ* and

(10) $${\text{h}}_{i}\left(\text{x}\right)={\text{I}}_{3,4}{\text{M}}_{\mu }^{-1}{\text{R}}_{\text{q}}^{-1}\frac{\partial {\text{R}}_{\text{q}}}{\partial q_{i}}{\text{M}}_{\mu }\left[\;\begin{array}{c} \text{x}\\ 1 \end{array}\right].$$

For a more stable algorithm, the matrix of second derivatives (Hessian matrix) should be positive definite. A suitable positive definite approximation to the Hessian (see Appendix A) is:

(11) $$\frac{\partial^{2}\varepsilon }{\partial q_{i}\partial q_{j}}≃\int_{\text{x}\in \Omega^{\prime }}\;w\left(\text{x}\right)\left(\text{g}\left(\text{x}\right)\cdot {\text{h}}_{i}\left(\text{x}\right)\right)\left(\text{g}\left(\text{x}\right)\cdot {\text{h}}_{j}\left(\text{x}\right)\right)d\text{x}.$$

Our implementation makes use of $\frac{\partial {\text{R}}_{\text{q}}}{\partial q_{i}}$, obtained by differentiating equation (3), although this could also have been computed numerically using finite differences or by more elegant methods (Al-Mohy and Higham, 2009). The overall optimization scheme is presented in Algorithm 1.

**Algorithm 1 |** Rigid-body registration.



**for** n=1...N **do**
  Initialize ${\text{q}}_{n}$.
**end for**
**repeat**
  Compute μ and ∇μ (equation 7).
  **for** n=1...N **do**
   Compute 1st derivatives (equation 9).
   Compute Hessian (equation 11).
   Gauss-Newton update of ${\text{q}}_{n}$ (equation 8).
  **end for**
  $\bar{\text{q}}\leftarrow \frac{1}{N}\sum_{n=1}^{N}{\text{q}}_{n}$.
  **for** n=1...N **do**
   ${\text{q}}_{n}\leftarrow {\text{q}}_{n}-\bar{\text{q}}$.
  **end for**
**until** convergence



### 2.3 Group-wise inhomogeneity correction

MRI scans are usually corrupted by a spatially smooth intensity non-uniformity (also known as “inhomogeneity” and sometimes referred to as “bias”), which is often corrected prior to image registration using a procedure such as N3 (Sled et al., 1998). Instead, we propose that the additional internal consistency from incorporating the non-uniformity correction within the longitudinal registration scheme should provide potentially more accurate results. Studholme et al. (2004), Modersitzki (2006), and Modat et al. (2010) previously used registration-based non-uniformity corrections, although those approaches did not consider aspects of inverse (or group-wise) consistency.

Here, we outline a general strategy for dealing with non-uniformity fields in aligned images. This part of the model assumes that a series of *N* aligned images (*f_n_*) may be modeled as a common template mean (*μ*), scaled by non-uniformity fields. Because these fields should be positive, they are modeled using an exponential of a Gaussian process $\left(e^{b_{n}}\right)$, where $b_{n}:\Omega_{f_{n}}\to \mathbb{R}$. This model leads to the following objective function.

(12) $$\varepsilon =\overset{N}{\underset{n=1}{\sum }}\left(\frac{\lambda_{n}}{2}{∥f_{n}-\mu e^{b_{n}}∥}^{2}+\frac{1}{2}{∥L_{b}b_{n}\;\;\;\;\;\;\;∥}^{2}\right)$$

Note that *L_b_* is a differential operator that penalizes the roughness of the (logarithms of the) estimated non-uniformity fields. A Laplacian is used in practice, although the optimal choice of differential operator will depend on the nature of the artifacts in the image data.

Regularized maximum likelihood (or maximum *a* *posteriori*) optimization of the inhomogeneity fields may be achieved by minimizing the above function. As in the rigid registration case, we propose alternating between re-estimating the mean and then using this to re-estimate the inhomogeneity fields.

The update of *μ* is achieved by differentiating equation (12) with respect to *μ*, and solving.

(13) $$\mu =\frac{\sum_{n=1}^{N}\;\lambda_{n}f_{n}e^{b_{n}}}{\sum_{n=1}^{N}\;\lambda_{n}e^{2b_{n}}}$$

Gradient descent could then be used to update the inhomogeneity fields, which requires the first derivatives of the objective function. These are computed via their Gâteaux differential.

(14) $$\begin{array}{l} d\varepsilon \left(b_{n};h\right)=\frac{d}{d\tau }½{\left(\lambda_{n}{\left\|f_{n}-\mu e^{b_{n}+\tau h}\right\|}^{2}+{\left\|L_{b}\left(b_{n}+\tau h\right)\right\|}^{2}\right)|}_{\tau =0}\\ \;=\int_{X\in \Omega_{\mu }}-a_{n}\left(\text{x}\right)\mu \left(\text{x}\right)h\left(\text{x}\right)d\text{x}+\langle L_{b}^{†}L_{b}L_{n},h\rangle \end{array}$$

where $a_{n}=\lambda_{n}e^{b_{n}}\left(f_{n}-\mu e^{b_{n}}\right).$

In our implementation, the fields are updated via a Gauss-Newton step, which makes use of a positive definite approximation to the second derivatives (derived using similar principles to those in Appendix A).

(15) $$\begin{array}{rcll} & d^{2}\varepsilon \left(b_{n};h_{1},h_{2}\right)=\frac{d^{2}}{d\tau_{1}d\tau_{2}}\frac{1}{2}\left(\lambda_{n}{∥f_{n}-\mu e^{b_{n}+\tau_{1}h_{1}+\tau_{2}h_{2}}∥}^{2}\right. & & \\ & \;{\left.+{∥L_{b}\left(b_{n}+\tau_{1}h_{1}+\tau_{2}h_{2}\right)∥}^{2}\right)\left|\right.}_{\tau_{1}=0,\tau_{2}=0} & & \\ & \;≃\int_{\text{x}\in \Omega_{\mu }}\;w_{n}\left(\text{x}\right)\mu {\left(\text{x}\right)}^{2}h_{1}\left(\text{x}\right)h_{2}\left(\text{x}\right)d\text{x}+\left\langle L_{b}^{†}L_{b}h_{1},h_{2}\right\rangle & \end{array}$$

where $w_{n}=\lambda_{n}e^{2b_{n}}.$

The overall procedure is summarized in Algorithm 2, which has a similar overall structure to Algorithm 1. The non-uniformity fields are encoded by a parameter at each voxel, such that continuous representations [of *b*(**x**)] may be obtained via tri-linear interpolation. This parameterization results in a diagonal matrix for the first term in equation (15), making it relatively straightforward to solve the system of linear equations required for the update via a full multi-grid (FMG) method.

**Algorithm 2 |** Non-uniformity field estimation.



**for** n=1...N **do**
  Initialize $b_{n}$.
**end for**
**repeat**
  Compute μ (equation 13).
  **for** n=1...N **do**
   Compute 1st derivatives (equation 14).
   Compute Hessian (equation 15).
   Gauss-Newton update of $b_{n}$ (via FMG).
  **end for**
  $\bar{b}\leftarrow \frac{1}{N}\sum_{n=1}^{N}b_{n}$.
  **for** n=1...N **do**
   $b_{n}\leftarrow b_{n}-\bar{b}$.
  **end for**
**until** convergence



Although the model appears to incorporate a number of un-necessary parameters (i.e., *N* non-uniformity fields plus a mean image), it effectively involves only *N* − 1 fields. The mean image is incorporated for convenience, and the geometric mean over all the estimated non-uniformity fields converges to one (given appropriate regularization). In practice, this constraint may be incorporated in the optimization (as shown in Algorithm 2), which enhances convergence. When there are two images, we may assume *b*_2_ = −*b*_1_, in which case the objective function reduces to

(16) $$\varepsilon =\frac{\lambda_{1}\lambda_{2}}{2}\int_{\text{x}\in \Omega }\frac{{\left(f_{1}\left(\text{x}\right)e^{b_{1}\left(\text{x}\right)}-f_{2}\left(\text{x}\right)e^{-b_{1}\left(\text{x}\right)}\right)}^{2}}{\left(\lambda_{1}e^{2b_{1}\left(\text{x}\right)}+\lambda_{2}e^{-2b_{1}\left(\text{x}\right)}\right)}d\text{x}+{∥L_{b}b_{1}∥}^{2}.$$

This special case may have other applications, such as the computation of smooth ratios of MR scans for mapping of RF transmit fields (Lutti et al., 2010).

### 2.4 Group-wise diffeomorphic registration

From a generative modeling perspective, the objective function for group-wise registration may be written as

(17) $$\varepsilon =\overset{N}{\underset{n=1}{\sum }}{\left(\frac{\lambda_{n}}{2}∥f_{n}-\mu \circ \phi_{{\text{v}}_{n}}^{-1}∥\right)}^{2}+\frac{1}{2}{∥{\text{L}}_{{\text{v}}_{n}}{\text{v}}_{n}∥}^{2}$$

where $\phi_{{\text{v}}_{n}}:\Omega_{\mu }\to \Omega_{\mu }$ is a diffeomorphic mapping, constructed from the initial velocity field ${\text{v}}_{n}:\Omega_{\mu }\to {\mathbb{R}}^{3}.$ Each image (*f_n_*) is assumed to be a warped version of a template (*μ*), with added noise. Smoothness of the diffeomorphisms are achieved via the differential operator **L_v_**, which may differ from image to image.

It is now reasonably well known that a diffeomorphism (**_v_**) may be computed from an initial velocity field (**v**) using a procedure known as geodesic shooting (Miller et al., 2006). Essentially, this procedure is based on integrating a particular form of dynamical system over unit time, and relies upon the principle of conservation of momentum. The procedure begins by initializing (**ϕ_v_**) to the identity transform and computing the initial momentum from the initial velocity via

(18) $$\text{u}={\text{L}}_{\text{v}}^{†}{\text{L}}_{\text{v}}\text{v.}$$

Then the following dynamical system is integrated over unit time.

(19) $${\overset{{}^{\circ}}{\phi }}_{\text{v}}=\left({\text{K}}_{\text{v}}\left(\left|\text{D}\phi_{\text{v}}^{-1}\right|{\left({\text{D}\phi }_{\text{v}}^{-1}\right)}^{T}\left(\text{u}\circ \phi_{\text{v}}^{-1}\right)\right)\right)\circ \phi_{\text{v}}$$

Briefly, the initial momentum is re-sampled according to the inverse of the current estimate of (**ϕ_v_**. Each point in the resulting field is matrix-multiplied by the transpose of the Jacobian tensor at that point of $\phi_{\text{v}}^{-1}$ and rescaled by the determinant of the Jacobian. Finally, the result is smoothed by applying the **K_v_** operator, which is the Green’s function of ${\text{L}}_{\text{v}}^{†}{\text{L}}_{\text{v}}$ (see Bro-Nielsen and Gramkow, 1996), to give the velocity field that provides the next update for (**ϕ_v_**. The **K_v_** operator may be viewed as a low pass filter, which is the (pseudo-) inverse of ${\text{L}}_{\text{v}}^{†}{\text{L}}_{\text{v}},$ such that ${\text{K}}_{\text{v}}{\text{L}}_{\text{v}}^{†}{\text{L}}_{\text{v}}\text{v}=\text{v}$. In practice, we use a slightly different integration scheme, which was described in Ashburner and Friston (2011).

We now re-write Equation (17) via integration by substitution.

(20) $$\begin{array}{rcll} \varepsilon = & \overset{N}{\underset{n=1}{\sum }}\frac{\lambda_{n}}{2}\int_{\text{x}\in \Omega_{\mu }}\left|\text{D}\phi_{{\text{v}}_{n}}\left(\text{x}\right)\right|{\left(f_{n}\circ \phi_{{\text{v}}_{n}}\left(\text{x}\right)-\mu \left(\text{x}\right)\right)}^{2}d\text{x} & & \\ & +\frac{1}{2}\overset{N}{\underset{n=1}{\sum }}{∥{\text{L}}_{{\text{v}}_{n}}{\text{v}}_{n}∥}^{2} & \end{array}$$

The template update equation is obtained by differentiating equation (20) with respect to *μ* and solving.

(21) $$\mu =\frac{\sum_{n=1}^{N}\;\lambda_{n}\left|\text{D}\phi_{{\text{v}}_{n}}\right|\left(f_{n}\circ \phi_{{\text{v}}_{n}}\right)}{\sum_{n=1}^{N}\;\lambda_{n}\left|\text{D}\phi_{{\text{v}}_{n}}\right|}$$

Registration is treated as an optimization procedure, using both first and second derivatives. The first Gâteaux differential is computed via

(22) $$d\varepsilon \left({\text{v}}_{n};\text{h}\right)=\int_{\text{x}\in \Omega_{\mu }}\;a_{n}\left(\text{x}\right)\text{g}\left(\text{x}\right)\cdot \text{h}\left(\text{x}\right)d\text{x}+\left\langle {\text{L}}_{{\text{v}}_{n}}^{†}{\text{L}}_{{\text{v}}_{n}}{\text{v}}_{n},\text{h}\right\rangle$$

where $a_{n}=\lambda_{n}\left|\text{D}\phi_{{\text{v}}_{n}}\right|\left(f_{n}\circ \phi_{{\text{v}}_{n}}-\mu \right).$ The template gradients (**g**) used for the registration are computed (see Appendix B) as follows.

(23) $$\text{g}=\frac{\sum_{n=1}^{N}\;\lambda_{n}\left|\text{D}\phi_{{\text{v}}_{n}}\right|\nabla \left(f_{n}\circ \phi_{{\text{v}}_{n}}\right)}{\sum_{n=1}^{N}\;\lambda_{n}\left|\text{D}\phi_{{\text{v}}_{n}}\right|}$$

A Gauss-Newton step is used to update the estimates of the initial velocity, which also requires a positive definite approximation to the second derivatives.

(24) $$\begin{array}{rcll} d^{2}\varepsilon \left({\text{v}}_{n};{\text{h}}_{1},{\text{h}}_{2}\right) & ≃\int_{\text{x}\in \Omega_{\mu }}\;w_{n}\left(\text{x}\right)\left(\text{g}\left(\text{x}\right)\cdot {\text{h}}_{1}\left(\text{x}\right)\right)\left(\text{g}\left(\text{x}\right)\cdot {\text{h}}_{2}\left(\text{x}\right)\right)d\text{x} & & \\ & \;+\left\langle {\text{L}}_{{\text{v}}_{n}}^{†}{\text{L}}_{{\text{v}}_{n}}{\text{h}}_{1},{\text{h}}_{2}\right\rangle & \end{array}$$

where $w_{n}=\lambda_{n}\left|\text{D}\phi_{{\text{v}}_{n}}\right|.$

Further practical details about the implementation may be found in Ashburner and Friston (2011), with some explanation of how the system of linear equations are solved via a full multi-grid approach in Ashburner (2007).

**Algorithm 3 |** Diffeomorphic registration.



**for** n=1...N **do**
  Initialize ${\text{v}}_{n}$.
**end for**
**repeat**
  **for** n=1...N **do**
   Compute $\phi_{{\text{v}}_{n}}$ and $\text{D}\;\phi_{{\text{v}}_{n}}$ from ${\text{v}}_{n}$ using
 geodesic shooting.
  **end for**
  Compute mean and gradients
 [equations (21) and (23)].
  **for** n=1...N **do**
   Compute 1st derivatives (equation 22).
   Compute Hessian (equation 24).
   Gauss-Newton update of ${\text{v}}_{n}$ (via FMG).
  **end for**
  $\bar{\text{u}}\leftarrow \frac{1}{N}\sum_{n=1}^{N}\left({\text{L}}_{{\text{v}}_{n}}^{†}{\text{L}}_{{\text{v}}_{n}}\right){\text{v}}_{n}$.
  **for** n=1...N **do**
   ${\text{v}}_{n}\leftarrow {\text{v}}_{n}-{\text{K}}_{{\text{v}}_{n}}\bar{\text{u}}$.
  **end for**
**until** convergence



### 2.5 Combining the components

**Figure 1:**
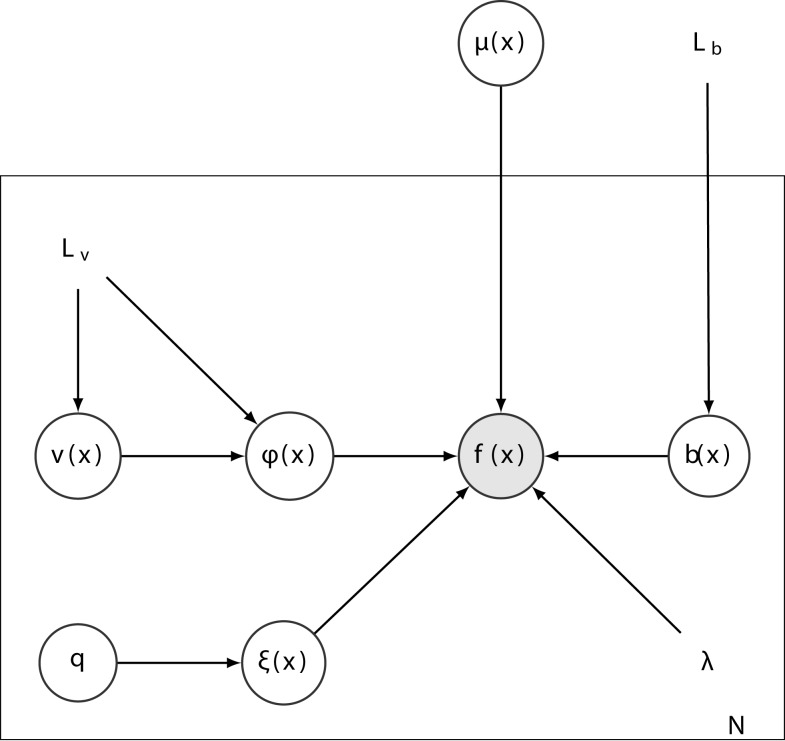
**A graphical representation of the full model**. Each of the *N* images (*f*) is assumed to be a deformed version of the template (*μ*) scaled by a multiplicative inhomogeneity field [exp(*b*)] with additive Gaussian noise (precision λ). Each deformation is modeled by the composition of a rigid-body transform (**ξ**) parameterized by a vector of six parameters (**q**), and a diffeomorphic deformation (***ϕ***) parameterized by its initial velocity (**v**).

The registration procedure combines group-wise rigid-body and diffeomorphic registration with intensity non-uniformity correction. The model assumes that each image (*f_n_*) is a deformed version of a template image (*μ*), scaled by the exponential of an inhomogeneity field (*b_n_*), with a known amount of additive i.i.d. Gaussian noise (precision λ*_n_*). Fitting this model (see Figure 1) involves minimizing the following objective function:

(25) $$\begin{array}{rcll} \varepsilon & =\overset{N}{\underset{n=1}{\sum }}\;\frac{1}{2}\left(\int_{\text{x}\in \Omega_{n}^{\prime }}\;\lambda_{n}\left|\text{D}\varphi_{n}\left(\text{x}\right)\right|{\left(f_{n}^{\prime }\left(\text{x}\right)-\mu \left(\text{x}\right)e^{b_{n}^{\prime }\left(\text{x}\right)}\right)}^{2}d\text{x}\right. & & \\ & \;\left.+{∥{\text{L}}_{{\text{v}}_{n}}{\text{v}}_{n}∥}^{2}+{∥L_{b}b_{n}∥}^{2}\right) & \end{array}$$

with the following definitions:

$$\begin{array}{rcll} & \varphi_{n}=\xi_{{\text{q}}_{n}}\circ \phi_{{\text{v}}_{n}} & & \\ & f_{n}^{\prime }=f_{n}\left(\varphi_{n}\right) & & \\ & b_{n}^{\prime }=b_{n}\left(\varphi_{n}\right) & & \\ & \Omega_{n}^{\prime }=\varphi_{n}^{-1}\left(\Omega_{f_{n}}\right)\cap \Omega_{\mu } & & \end{array}$$

The images (*f*) and template (*μ*) are treated as continuous functions, but are actually encoded via B-spline basis functions. This is done to achieve continuous spatial gradients, which are needed for the registration. Velocity (**v**) and logarithms of inhomogeneity fields (*b*) are also treated as spatially continuous, and represented using tri-linear interpolation.

We now introduce a few more definitions that will be useful later. The mean image is needed for all steps, and is computed by

(26) $$\mu =\frac{\sum_{n=1}^{N}\;w_{n}e^{-b_{n}^{\prime }}f_{n}^{\prime }}{\sum_{n=1}^{N}\;w_{n}},$$

where

(27) $$w_{n}\left(\text{x}\right)=\left\{\begin{array}{cc} \lambda_{n}\left|\text{D}\varphi_{n}\left(\text{x}\right)\right|e^{2b_{n}^{\prime }}\left(\text{x}\right)\; & \text{if}\;\text{x}\;\in \;\Omega_{n}^{\prime }\\ 0\; & \text{otherwise}\\ \; \end{array}\right.$$

In addition, the following gradients are required for driving the image registration

(28) $$\begin{array}{llll} \text{g} & =\frac{\sum_{n=1}^{N}\;w_{n}e^{-b_{n}^{\prime }}\left(\nabla f_{n}^{\prime }+f_{n}^{\prime }\nabla b_{n}^{\prime }\right)}{\sum_{n=1}^{N}\;w_{n}} & & \\ & \;-\frac{2\left(\sum_{n=1}^{N}\;w_{n}e^{-b_{n}^{\prime }}f_{n}^{\prime }\right)\left(\sum_{n=1}^{N}\;w_{n}\nabla b_{n}^{\prime }\right)}{{\left(\sum_{\text{n}=1}^{n}\;w_{n}\right)}^{2}}. \end{array}$$

One other definition is

(29) $$a_{n}=w_{n}\left(f_{n}^{\prime }e^{-b_{n}^{\prime }}-\mu \right).$$

The derivatives used for updating the rigid-body and diffeomorphic transformations are computed by substituting the above expressions. To compute the first derivatives, equations (28) and (29) would be substituted into equations (9) and (22). For the approximate second derivatives, equations (27) and (28) would be substituted into equations (11) and (24).

The main difficulty arises from combining the inhomogeneity estimation with the registration. Others have used an elegant metamorphosis approach (Trouvé and Younes, 2005) for integrating intensity variations with deformations, but this is not appropriate here. In the current model, intensity variations are assumed to be a function of the MR scanner rather than an intrinsic property of the brain itself. Therefore, the inhomogeneity fields are estimated in the space of the original images. Our registration scheme involves estimating mappings from the mean template to the individual scans. We can re-sample the original scans to bring them in alignment with the template, but sampling the template to align it with the individual scans would require additionally computing the inverse deformation, adding an additional level of computational complexity. Our solution is to first compute derivatives of the data term in the space of the template, making use of the Jacobians of the deformations in a substitution of variables.

**Algorithm 4 |** Combined model estimation.



**for** n=1...N **do**
  Initialize ${\text{q}}_{n}$, $b_{n}$ and ${\text{v}}_{n}$.
**end for**
**repeat**
  **for** n=1...N **do**
   Compute $\phi_{{\text{v}}_{n}}$ and $\text{D}\phi_{{\text{v}}_{n}}$ from ${\text{v}}_{n}$
 (see Ashburner and Friston 2011).
  **end for**
  Re-compute μ and **g**
 [equations (26) and (28)].
  **for** n=1...N **do**
    Compute $\frac{\partial \varepsilon }{\partial q}$ (equation 9,
 using equation 29).
   Compute $\frac{\partial^{2}\varepsilon }{\partial q^{2}}$ (equation 11,
 using equation 27).
    Gauss-Newton update of ${\text{q}}_{n}$
 (equation 8).
  **end for**
   $\bar{\text{q}}\leftarrow \frac{1}{N}\sum_{n=1}^{N}{\text{q}}_{n}$.
  **for** n=1...N **do**
    ${\text{q}}_{n}\leftarrow {\text{q}}_{n}-\bar{\text{q}}$.
  **end for**
  Re-compute μ (equation 26).
  **for** n=1...N **do**
    Compute $d\varepsilon \left(b_{n};h\right)$ (equation 30, using
 equation 29).
   Compute $d^{2}\varepsilon \left(b_{n};h_{1},h_{2}\right)$ (equation 31,
 using equation 27).
    Gauss-Newton update of $b_{n}$.
  **end for**
  Re-compute μ and g (equations 26 and 28).
  **for** n=1...N **do**
   Compute $d\varepsilon \left({\text{v}}_{n};\text{h}\right)$ (equation 22,
 using equation 27).
   Compute $d^{2}\varepsilon \left({\text{v}}_{n};{\text{h}}_{1},{\text{h}}_{2}\right)$ (equation 24,
 using equation 27).
    Gauss-Newton update of ${\text{v}}_{n}$.
  **end for**
   $\bar{\text{u}}\leftarrow \frac{1}{N}\sum_{n=1}^{N}\left({\text{L}}_{{\text{v}}_{n}}^{†}{\text{L}}_{{\text{v}}_{n}}\right){\text{v}}_{n}$.
  **for** n=1...N **do**
   ${\text{v}}_{n}\leftarrow {\text{v}}_{n}-{\text{K}}_{{\text{v}}_{n}}\bar{\text{u}}$.
  **end for**
**until** convergence



Equations (14) and (15) may be re-written to incorporate equations (27) and (29).

(30) $$\begin{array}{l} \;d\varepsilon \left(b_{n};h\right)=\int_{\text{x}\in \Omega f_{n}}-\left|\text{D}\phi_{\text{n}}^{-1}\left(\text{x}\right)\right|\left(a_{n}\mu \right)\circ \phi_{n}^{-1}\left(\text{x}\right)h\left(\text{x}\right)d\text{x}\\ \;+\langle L_{b}^{†}L_{b}b_{n},h\rangle \end{array}$$

(31) $$\begin{array}{l} d^{2}\varepsilon \left(b_{n};h_{1},h_{2}\right)≃\int_{\text{x}\in \Omega f_{n}}\left|\text{D}\phi_{\text{n}}^{-1}\left(\text{x}\right)\right|\left(w_{n}\mu^{2}\right)\circ \phi_{n}^{-1}\left(\text{x}\right)\\ \;h_{1}\left(\text{x}\right)h_{2}\left(\text{x}\right)d\text{x}+\langle L_{b}^{†}L_{b}h_{1},h_{2}\rangle \end{array}$$

This enables the derivatives to be first computed in the space of the template, and subsequently pushed forward to the space of the original images. The inhomogeneity fields are then re-estimated by an iteration of Gauss-Newton, although they are not mean-corrected.

The overall optimization scheme is shown in Algorithm 4.

### 2.6 Implementation details

Our implementation is written in a mixture of MATLAB and C code (mex files for the computationally expensive parts). For additional speed (and accuracy), the overall procedure is run over multiple spatial scales, beginning at the lowest resolution. At each scale, a solution is computed, which is prolonged to the next scale where it serves as a starting estimate for the next set of iterations at a higher resolution.

The implementation has large memory requirements, which are likely to exceed the addressable memory of 32-bit computers. To save some memory, many of the computations are done using single precision floating point. Briefly, the main memory consumption comes from:

- Image data (*f_n_*). If each image contains *J* voxels, the memory for all images will be 4*JN* bytes. For example, a 256 × 256 × 256 image (where *J* = 16777216) requires 64 MB to represent it as single precision floating point.
- Inhomogeneity fields (*b_n_*) require 4*JN* bytes.
- Template (*μ*) and its spatial gradients (**g**) require 4*J_μ_* + 12*J_μ_* bytes, where *J_μ_* is the number of voxels in the template.
- Velocity fields (**v***_n_*) require 12*J_μ_N* bytes.
- Deformation fields $\left(\phi_{{\text{v}}_{n}}\right)$ require 12*J_μ_N* bytes.
- Jacobian fields $\left(\text{D}\phi_{{\text{v}}_{n}}\right)$ require 36*J_μ_N* bytes.

The geodesic shooting step consumes a lot of additional memory. This includes 36*J_μ_* bytes for the Fourier transform of the Green’s function (**K**), 12*J_μ_* bytes for the initial momentum (**u***_n_*), plus 96*J_μ_* bytes for composing diffeomorphisms and their Jacobians. The maximum requirement at any point is just over 144*J_μ_* + *N*(8*J* + 60*J_μ_*) bytes.

The algorithm requires a few user-defined settings, which will now be outlined.

#### 2.6.1 Noise estimates

The model assumes that images can be closely aligned, such that most of the residual variance is scanner noise. One of the settings needed by the algorithm is an estimate of the noise variance of each scan $\left(\frac{1}{\lambda_{n}}\right)$. This may be assigned by the user, although our implementation defaults to using values estimated from the MR scans themselves.

**Figure 2:**
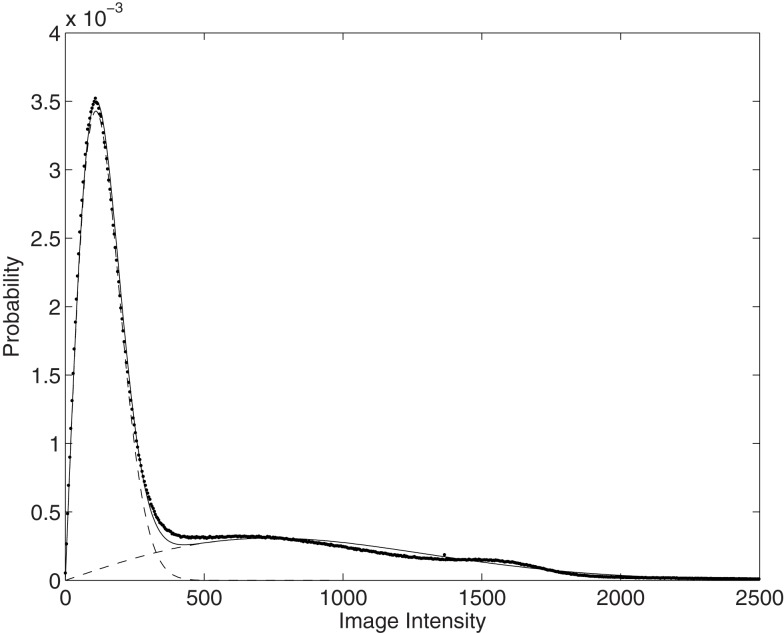
**A mixture of two Rician distributions fit to an MRI intensity histogram (shown dotted)**. The fit is shown as a continuous line, whereas dashed lines are used to show the two Rician distributions.

Because MR images usually encode the magnitude of complex data, they have Rician noise. An estimate of the variance of this noise can be made by fitting a mixture of two Rician distributions to an intensity histogram from an image (see Figure 2). A reasonable scanner noise estimate may then be obtained from the smaller of the two estimated variances. The fitting procedure is similar to the expectation maximization for fitting a mixture of Gaussians, although the SNR fixed point formula (Koay and Basser, 2006) is used to compute the Rician parameters from the sample means and standard deviations.

Noise drawn from a Rician distribution deviates most from Gaussian in regions of low signal intensity, where it is closer to the Rayleigh distribution (Rician with zero signal). Although the mean-squares difference noise model (which assumes residuals are drawn from a Gaussian distribution) differs from the Rician assumptions, it is probably close enough within the regions of the image in which we are mostly interested. Even in the worst case, the difference between noise drawn from two Rayleigh distributions is close to Gaussian.

#### 2.6.2 Differential operator for inhomogeneity

Without regularization, all the differences among the images would be explained by the estimated inhomogeneity fields. Therefore, their estimation needs to involve regularization, which will depend on the nature of the artifacts present. If there is no inhomogeneity, then very strong regularization may be used, whereas less would be used in the presence of large artifacts. Our implementation regularizes by minimizing the bending energy of the fields $({∥L_{b}b∥}^{2}=\omega_{0}\int_{\text{x}\in \Omega }\;{∥\nabla^{2}b\left(\text{x}\right)∥}^{2}d\text{x}$, where ω_0_ is a hyper-parameter controlling the strength of the regularization), which gives generally smooth estimates. Neumann boundary conditions are imposed on *b*, such that the gradient is zero at the edge of the field of view.

#### 2.6.3 Differential operator for diffeomorphisms

The solution of any image registration problem is heavily dependent on the choice of differential operator used to regularize it. Although there are principled (Bayesian) ways to optimize the operator (Simpson et al., 2011), such a strategy would be beyond the scope of the current work. Instead, a relatively *ad hoc* choice about the form of operator was made, although there were still some principled aspects involved.

The differential operator involved no penalty against absolute displacements, which has implications for the Green’s function (**K_v_**) used by the geodesic shooting procedure. Such forms of regularization operator can not be inverted exactly to obtain a unique **K_v_**. Our implementation of geodesic shooting used Fast Fourier Transform (FFT) methods to obtain the Green’s function. Because the DC coefficient of the FFT of the differential operator is zero, its reciprocal is infinity. We therefore set the DC coefficient of the Green’s function to zero, and let the rigid-body registration account for global translations.

Many registration approaches use a Green’s function that is simply a Gaussian. We chose to avoid this, as such functions privilege certain spatial scales above others (and also penalize absolute displacements). Some have instead used a **K_v_** consisting of a mixture of Gaussians to account for multiple spatial scales (Risser et al., 2011). Our approach involves using a combination of the linear-elasticity and bending energy (or thin-plate) models.

(32) $$\begin{array}{rcll} {∥{\text{L}}_{\text{v}}\text{v}∥}^{2} & =\int_{\text{x}\in \Omega }\;\left(\frac{\omega_{1}}{4}{∥\text{Dv}\left(\text{x}\right)+{\left(\text{Dv}\left(\text{x}\right)\right)}^{T}∥}_{F}^{2}+\omega_{2}\text{tr}{\left(\text{Dv}\left(\text{x}\right)\right)}^{2}\right. & & \\ & \left.\;+\omega_{3}{∥\nabla^{2}\text{v}\left(\text{x}\right)∥}^{2}\right)d\text{x} & \end{array}$$

Three hyper-parameters are involved:

- *ω*_1_ controls the amount of stretching and shearing (but not rotation).
- *ω*_2_ controls the divergence, which in turn determines the amount of volumetric expansion and contraction.
- *ω*_3_ controls the bending energy. This ensures that the resulting velocity fields have smooth spatial derivatives.

The Neumann boundary condition could not be used for the velocity fields, so these are assumed to be circular (the same as for a Fourier transform).

#### 2.6.4 Acquisition timing adjustments

When there are only two scans to align, it is natural to encode the template at the point half-way between them. However, when the number of scans is greater than two, the choice of what time-point the template corresponds to is more arbitrary. If the regularization is the same for aligning all scans with the template, then the natural point is the average time of all scans. Generally, we expect larger deformations for situations where the time interval between the template and scan is greater. Therefore, the regularization is adjusted for each scan so that it is (approximately) inversely proportional to the absolute time difference between the template and scan acquisition time. We also assume that the time for the template corresponds to the median of the acquisition times. Accounting for this requires the warping regularization to be adjusted, such that the penalty is in terms of an energy measure per unit of time. If the interval between template and scan is *t_n_* units (e.g., years), the penalty for the deformation is defined as $\frac{1}{2}{∥{\text{L}}_{{\text{v}}_{n}}{\text{v}}_{n}∥}^{2}=\frac{1}{2|t_{n}|}{∥{\text{L}}_{\text{v}}{\text{v}}_{n}∥}^{2}$, where **L_v_** encodes the penalty for one unit of time difference.

#### 2.6.5 Integrations

The equations within the Methods section describe a continuous setting, whereas our actual implementation replaces the integrations over space with summations sampling the voxel centers. This sort of approximation is widely used for image registration, although it probably accounts for much of the findings in Yushkevich et al. (2010).

The integrations over time, which are used by the geodesic shooting procedure, also need to be discretized. These are currently done using an Euler integration scheme, which uses three steps per unit of time difference, plus an additional two steps to account for some of the larger distortions usually found in the soft tissue outside the skull. Faster registration could be achieved using fewer time steps, although this may lead to decreased accuracy.

## 3 Results

It is difficult to evaluate models when there is no ground truth available. Although in theory, Bayesian model comparisons could be performed, these would require computations that are not currently feasible for very large models. Therefore, the evaluations are mostly anecdotal.

### 3.1 Effects of regularization

**Figure 3:**
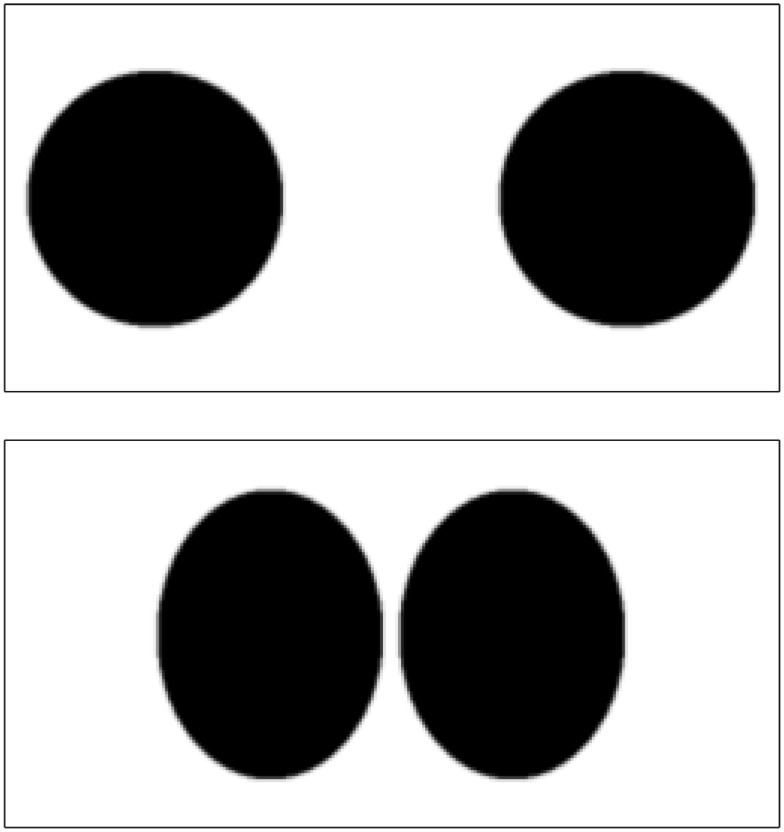
**The two simulated images**.

**Figure 4:**
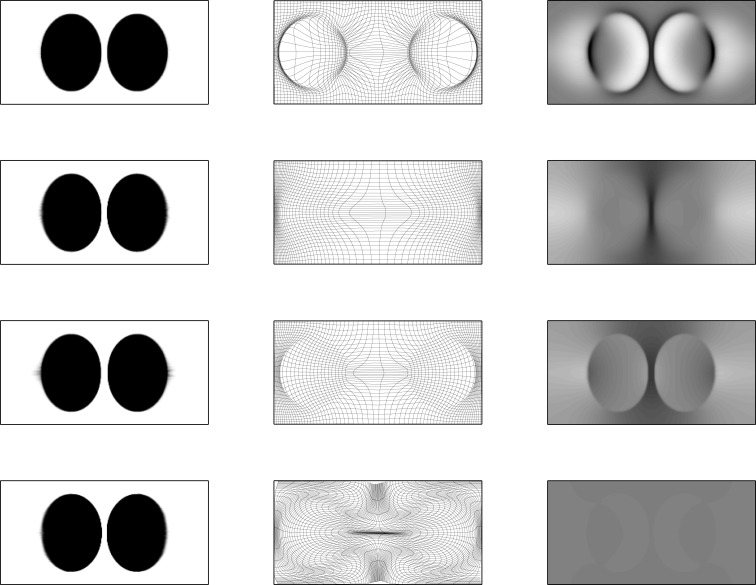
**Warped simulated images**. First column: upper image warped to match lower image (from Figure 3). Second column: deformation fields. Third column: logarithms of Jacobian determinants (color-scales are the same for all examples, and in the range of −3 to 3). First row: results from ω_1_ = 0.001, ω_2_ = 0.001, and ω_3_ = 0.1, with an additional penalty on the square of absolute displacements of 0.0001. Second row: results from ω_1_ = 0.001, ω_2_ = 0.001, and ω_3_ = 2.0. Third row: results from ω_1_ = 0.05, ω_2_ = 0.0001, and ω_3_ = 0.0001. Fourth row: results from ω_1_ = 0.001, ω_2_ = 0.5, and ω_3_ = 0.001.

**Figure 5:**
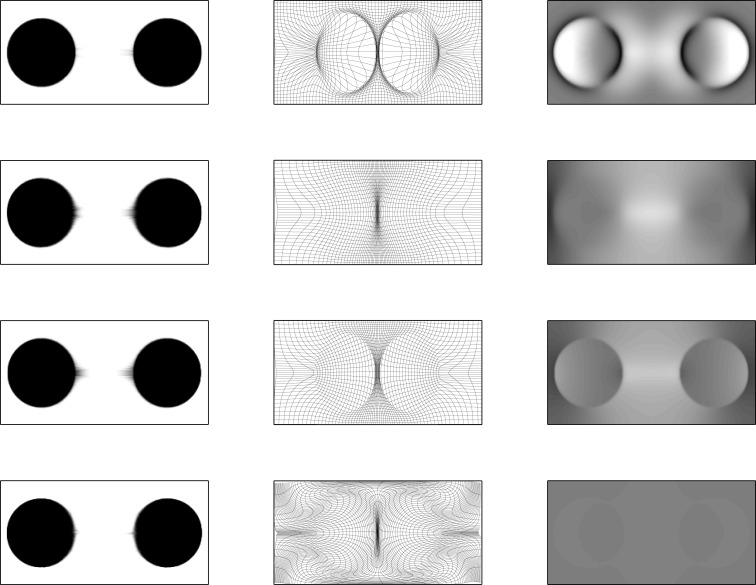
**Warped simulated images**. First column: lower image warped to match upper image. Second column: deformation fields. Third column: logarithms of Jacobian determinants (color-scales are the same for all examples, and in the range of −3 to 3). First row: results from ω_1_ = 0.001, ω_2_ = 0.001, and ω_3_ = 0.1, with an additional penalty on the square of absolute displacements of 0.0001. Second row: results from ω_1_ = 0.001, ω_2_ = 0.001, and ω_3_ = 2.0. Third row: results from ω_1_ = 0.05, ω_2_ = 0.0001, and ω_3_ = 0.0001. Fourth row: results from ω_1_ = 0.001, ω_2_ = 0.5, and ω_3_ = 0.001.

The effects of regularizing diffeomorphisms using different choices of penalty is demonstrated using simulated 2D data. This involves the two images shown in Figure 3, which have dimensions of 256 × 128 pixels, and intensities ranging from 0 to 1. The areas of the circles and ellipses in the simulation were all approximately the same. These were registered together using the diffeomorphic framework – but without intensity inhomogeneity correction – using a variety of different forms of regularization. Results are shown in Figures 4 and 5. The first thing to notice is that the warped images all look relatively similar to each other, but the deformations (and their Jacobian determinants) differ markedly. The choice of regularization will play a significant role in any study where the aim is to localize volumetric differences.

1. The first form of regularization (*ω*_1_ = 0.001, *ω*_2_ = 0.001, and *ω*_3_ = 0.1, with an additional penalty on the square of absolute displacements of 0.0001) is intended to demonstrate the effect of using a Green’s function that is approximately Gaussian. The behavior of such a kernel is such that the Jacobians of the resulting deformations are more extreme. For real longitudinal data in Alzheimer’s disease, where ventricles expand over time, it will give the impression that brain atrophy is localized to the regions close to the ventricles.
2. The second form (*ω*_1_ = 0.001, *ω*_2_ = 0.001, and *ω*_3_ = 2.0) is dominated by a penalty against the bending energy of the deformations. For expanding ventricles, it may give the impression that brain tissue around the ventricles also expands. In general though, this form of regularization has a number of nice properties, which include scale invariance.
3. The third form is dominated by the penalty against length changes. Unfortunately, within a continuous framework, the Green’s function for this one is sharply peaked, such that the value at the center is infinity.
4. The fourth form predominantly penalizes the divergence of the velocity fields, which tends to push the Jacobian determinants toward a value of one. In the bottom right of Figures 4 and 5, we see that the estimated volumetric differences are very small. The pure form of this regularization also has a Green’s function with a singularity.

The optimal form of regularization is likely to involve a combination of the above. Although liable to have a large impact on the accuracy of image registration algorithms, the neuroimaging literature contains little on the subject.

### 3.2 Real longitudinal MRI

The algorithm was evaluated using data downloaded from Part 1 of the *OASIS Longitudinal MRI Data in Non-demented and Demented Older Adults*^6^ dataset (Marcus et al., 2010). This contained longitudinal scans from 82 subjects (from OAS2_0001 to OAS2_0099), each with data from between two and five time-points. Data from each time-point consisted of between two and five MRI scans, permitting improved signal to noise ratios via averaging. Further demographic information about the subjects may be obtained from the OASIS web site.

The first step of the processing was to create averages of the scans from each time-point. Because subjects may move slightly, these averages were computed after a group-wise rigid-body alignment. This was achieved using the estimated template from the group-wise registration – but with non-linear deformations disabled.

The evaluations were based on averages over a number of scans, where the noise is no longer Rician. To simulate typical user behavior (default settings), they were done using noise estimates obtained by fitting Rician distributions to non-Rician noise.

#### 3.2.1 Pairwise symmetry

The first test was to assess whether the procedure is actually inverse consistent if run pairwise. This simply involved aligning the first and second time-points of a pair of longitudinal images, and assessing whether the results were compatible with those from aligning the second and first. They were found to be exactly consistent.

#### 3.2.2 Anecdotal example 1

**Figure 6:**
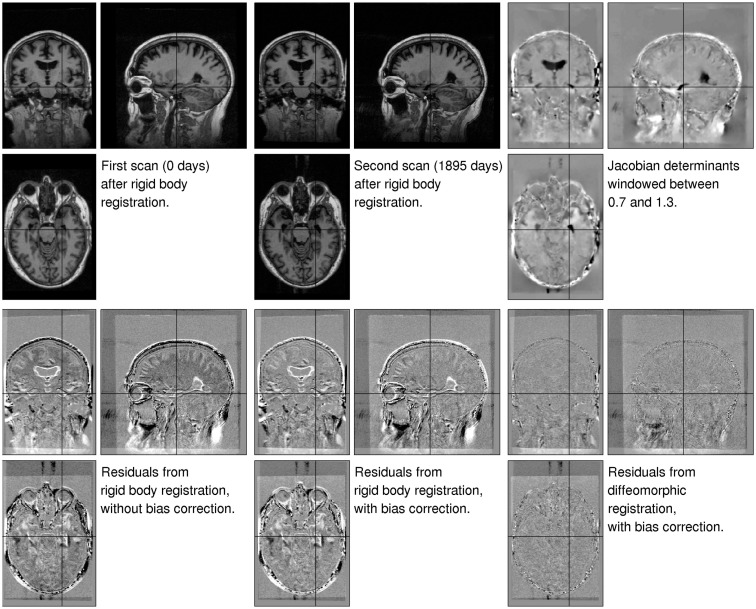
**Illustration of the results obtained from matching a pair of images of a subject with mild cognitive impairment, which were collected 1895 days apart (OAS2_0002)**. The three images of the residual difference shown along the bottom are all windowed the same. Black indicates a value of −500 or less, whereas white indicates values of 500 or above.

**Figure 7:**
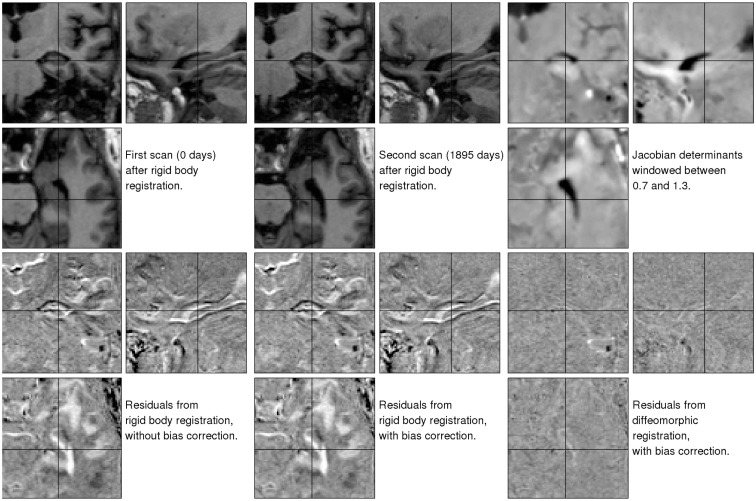
**Detail of the results obtained from matching a pair of images of a subject with mild cognitive impairment, which were collected 1895 days apart (OAS2_0002)**.

The first illustration uses data from a 75-year-old (at first scan) right handed male with mild cognitive impairment (OAS2_0002, MMSE = 22, CDR = 0.5). Although there were three images for this subject, we just ran the algorithm using the first and last, which were collected 1869 days apart. The primary aim was to show the decrease in residual difference, after both inhomogeneity correction and registration. This provides an indication of how well the registration works, but does not give the full story (Rohlfing, 2012) because some very implausible deformations may also greatly reduce the residuals. Jacobian determinant maps are also shown, which tell us about the plausibility of the volumetric changes involved. These results are shown in Figure 6, with more detail around the right hippocampus shown in Figure 7.

The first thing to note is that the registration greatly reduced the residual difference. After registration, there is little remaining structure to be seen in the residuals – particularly within the brain. We note also that the estimated Jacobian determinants seem to be plausible. A comparison between the residuals depicted in the bottom center and bottom left of the Figures shows the effect of the inhomogeneity correction, which also reduces the residual difference for these data.

The brain is enclosed within the skull, so there is relatively little external influence on its shape. This may be contrasted with the soft tissue outside the skull, which shows extensive shape changes due to the subject’s head positioning within the scanner. The Jacobian images (Figure 6) also show some artifacts along the right hand edge. These result from the image data wrapping around (see close to the right hand edge on the coronal view of the second time-point image).

#### 3.2.3 Anecdotal example 2

**Figure 8:**
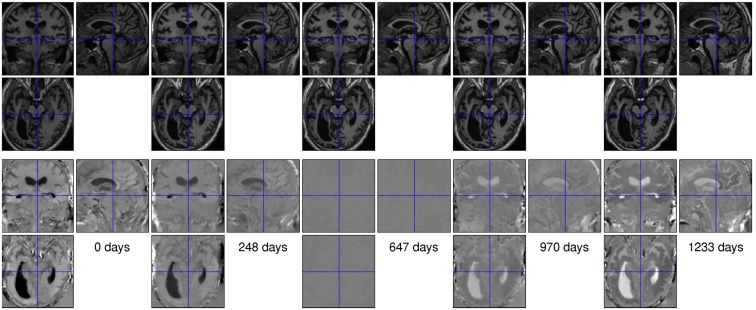
**Data from the time-points of a subject with dementia (OAS2_0048)**. The top row shows the original scans after rigid alignment, whereas the bottom row shows the divergence of the estimated velocity fields.

The second illustration uses data from a 66-year-old male with dementia (OAS2_0048, MMSE = 19, CDR = 1). There were five scans for this subject, collected over a period of 1233 days. Rigidly aligned versions of the images are shown along the top of Figure 8, with the corresponding maps of expansion (divergence of initial velocity) shown below. Note that the expansion map of the middle time-point is almost zero, as that point served as the reference time for the group-wise alignment. Careful examination of the divergence maps also reveals what appear to be artifactual volume changes for the more prominent blood vessels. This effect was found in many of the subjects.

#### 3.2.4 Principal components

**Figure 9:**
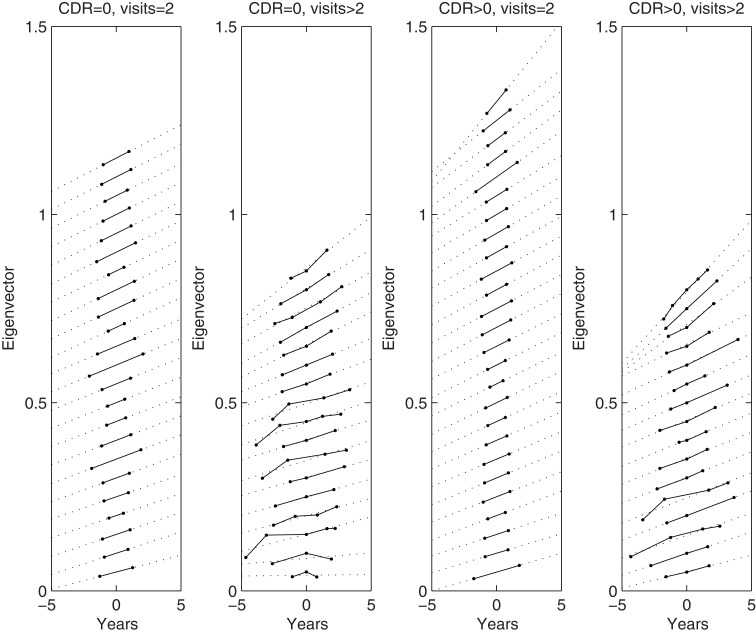
**Plots of main eigenvector from each subject’s divergence maps within the cranium**. The columns show plots from those control subjects who were scanned only twice, plots from control subjects who were scanned more than twice, plots from subjects with dementia who were scanned twice, and plots from subjects with dementia who were scanned more than twice. Dotted lines show the best linear fit. Note that the plots are sorted according to their average slope, which was done for easier visualization. Some of the eigenvectors were also rescaled by −1, such that all the gradients are positive.

Group-wise longitudinal registration was run for all 82 subjects’ data. The region within the cranium of each subject was identified by running the “new segment” algorithm of SPM8 (Ashburner and Friston, 2005) on their aligned mean images (*μ*), and summing the estimated gray matter, white matter and CSF maps together. Divergence values from inside the cranium of each subject were collected, from which *N* × *N* Gram matrices were computed and normalized by the number of voxels. The Gram matrices were decomposed via an eigen-decomposition and the largest eigenvalues identified. The corresponding eigenvectors, scaled by the square root of the eigenvalues, are plotted in Figure 9.

In general, the plots for multiple time-points appeared fairly linear, although we do notice a steeper gradient between the first two points for some of the subjects. Although it appears to be some form of artifact, we do not yet have a good explanation for it.

### 3.3 Mean images

Images of mean expansion rate were computed for each subject, by fitting voxel-wise linear models through the divergence maps.

A mapping between each subject’s gray matter, white matter, and CSF and the population mean of these tissues was estimated using Dartel (Ashburner, 2007; Ashburner and Friston, 2009). In addition, an affine mapping between the population mean and MNI space was also estimated. The compositions of these mappings were then used to warp each subject’s mean (*μ*) and their expansion rate images to MNI space^7^.

**Figure 10:**
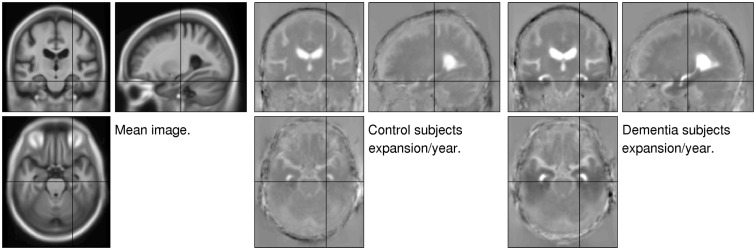
**Mean images**. Left: average of all subjects’ warped mean images. Center: average of the warped expansion rate maps of the control subjects. Right: average of the warped expansion rate maps of the subjects with dementia. Mean expansion rates are shown such that values of −0.04 or below are shown as black and values of 0.04 or above are shown as white.

A simple average (not weighted by Jacobian determinants) of all the warped mean images was computed. Similarly, simple averages of the warped expansion rate maps for the control subjects and subjects with dementia were also computed. These averages are shown in Figure 10, and clearly show the pattern of atrophy typically found in aging and dementia. Outside the brain, age associated skin thickness decreases can also be seen (Shuster et al., 2006).

## 4 Discussion

Research in biology is about much more than collecting p-values. Ultimately, we want to understand the mechanisms behind brain growth, development, aging, and various disease processes. Mechanistic models require internal consistency and plausible underlying assumptions. When internal consistency is not achieved, it is an indication that something is wrong. We demonstrated that for pairwise registration, our approach gives consistent solutions – irrespective of the order of the images.

Mechanistic models should be based ideally on well accepted underlying assumptions, which for image registration are that lengths, areas, and volumes should never fall below zero. Relative volumes are computed from deformations via the Jacobian determinants, so a necessary condition for a valid growth model is that these Jacobian determinants must be positive. Achieving this requires a diffeomorphic deformation framework.

Some readers may object to the use of large deformation diffeomorphic registration^8^ approaches for modeling the relatively small longitudinal changes seen in aging. In principle though, a related framework would also be applicable to modeling growth and development of the brain – or indeed any other organ – from fetus through to adult. Small-deformation approximations would fail for these more extreme changes, whereas a diffeomorphic approach could potentially model volumes and lengths that change by orders of magnitude.

Further modifications to the current approach would be required to account for intensity changes that are intrinsic to the brain. For example, the brains of young infants have changing appearances throughout myelogenesis. Similarly, white matter hypo-intensities, stroke, etc., may be more properly explained by intensity changes rather than shape changes, although it is not always entirely clear what solution is more appropriate. Others are beginning to develop models for simultaneous shape and appearance changes (Trouvé and Younes, 2005; Hong et al., 2012), although there is still much more to be done. Alternatively, greater robustness may be achieved by using a matching term other than the L^2^ norm, for which residual differences ideally follow a Gaussian distribution. A distribution with fatter tails, such as a mixture of Gaussians, may be more appropriate for modeling outliers (Penny et al., 2007).

The effects of changing the form and magnitude of the regularization are still relatively unexplored. It is likely that the optimal amount will depend on the ultimate objectives of a study. More regularization will decrease the noise in the estimated deformations – at the expense of introducing bias. Less regularization will decrease the bias, at the expense of fitting noise. If the aim is to compute volume changes of brain structures – or even whole brains – it may be better to use less regularization because the noise will be averaged out over multiple voxels. However, if the aim is to make use of values in individual voxels, the optimal bias-variance tradeoff will be achieved with heavier regularization.

We have presented a generative modeling framework for longitudinal MRI, which combines rigid alignment, diffeomorphic warping and differential intensity non-uniformity correction with respect to a within-subject template that evolves to be an average with regard to all three of these aspects. The approach is symmetric and transitive by construction. In the pairwise case, it is not only inverse consistent, but the path (on the manifold of diffeomorphisms) from one image to the other via the template is consistent with the direct geodesic path between the images. However, there is scope for further refinement of the model when dealing with images collected at more than two time-points. Such extensions would require ideas about variable rates of growth to be incorporated (Fishbaugh et al., 2011; Niethammer et al., 2011; Trouvé and Vialard, 2012) and will be investigated in future work.

## Conflict of Interest Statement

The authors declare that the research was conducted in the absence of any commercial or financial relationships that could be construed as a potential conflict of interest.

## Appendix

### A. Derivatives for rigid-body registration

To differentiate equation (5), we consider a single image and drop the *n* subscripts.

(A1) $$\frac{\lambda }{2}\int_{\text{x}\in \Omega_{f}}\;\left(\frac{\partial {\left(f-\mu \left(\xi_{\text{q}}^{-1}\right)\right)}^{2}}{\partial q_{i}}\right)d\text{x}=\lambda \int_{\text{x}\in \Omega_{f}}\;\left(\mu \left(\xi_{\text{q}}^{-1}\right)-f\right)\left(\frac{\partial \xi_{\text{q}}^{-1}}{\partial q_{i}}\cdot \left(\left(\nabla \mu \right)\circ \xi_{\text{q}}^{-1}\right)\right)d\text{x}.$$

Then a change of variables is incorporated to obtain the following:

(A2) $$\lambda \int_{\text{x}\in \Omega^{\prime }}\;\left|\text{D}\xi_{\text{q}}\right|\left(\left(\left(\mu \left(\xi_{\text{q}}^{-1}\right)-f\right)\left(\frac{\partial \xi_{\text{q}}^{-1}}{\partial q_{i}}\cdot \left(\left(\nabla \mu \right)\circ \xi_{\text{q}}^{-1}\right)\right)\right)\circ \xi_{\text{q}}\right)d\text{x}=\lambda \int_{\text{x}\in \Omega^{\prime }}\;\left|\text{D}\xi_{\text{q}}\right|\left(\mu -f\left(\xi_{\text{q}}\right)\right)\left(\left(\frac{\partial \xi_{\text{q}}^{-1}}{\partial {\text{q}}_{i}}\circ \xi_{\text{q}}\right)\cdot \left(\nabla \mu \right)\right)d\text{x}.$$

Computing the above requires $\frac{\partial \xi_{\text{q}}^{-1}}{\partial q_{i}}\circ \xi_{\text{q}}.$ Inverting equation (4) gives:

(A3) $$\xi_{\text{q}}^{-1}\left(\text{x}\right)={\text{I}}_{3,4}{\text{M}}_{\mu }^{-1}{\text{R}}_{q}^{-1}\text{M}\left[\;\begin{array}{c} \text{x}\\ 1 \end{array}\right].$$

Using the identity $\frac{\partial \left({\text{R}}^{-1}\right)}{\partial q}=-{\text{R}}^{-1}\frac{\partial \text{R}}{\partial q}{\text{R}}^{-1}$ to differentiate this, results in:

(A4) $$\frac{\partial \xi_{\text{q}}^{-1}}{\partial q_{i}}=-{\text{I}}_{3,4}{\text{M}}_{\mu }^{-1}{\text{R}}_{\text{q}}^{-1}\frac{\partial {\text{R}}_{\text{q}}}{\partial q_{i}}{\text{R}}_{\text{q}}^{-1}\text{M}\left[\;\begin{array}{c} \text{x}\\ 1 \end{array}\right].$$

This is combined with equation (4) to generate:

(A5) $$\frac{\partial \xi_{\text{q}}^{-1}}{\partial q_{i}}\circ \xi_{\text{q}}=-{\text{I}}_{3,4}{\text{M}}_{\mu }^{-1}{\text{R}}_{\text{q}}^{-1}\frac{\partial {\text{R}}_{\text{q}}}{\partial q_{i}}{\text{M}}_{\mu }\left[\;\begin{array}{c} \text{x}\\ 1 \end{array}\right].$$

Gauss-Newton optimization requires a positive definite approximation to the matrix of second derivatives equation (11). The actual Hessian matrix is given by the following, but it is not guaranteed to be positive definite:

(A6) $$\frac{\partial^{2}}{\partial q_{i}\partial q_{j}}\frac{\lambda }{2}\int_{\text{x}\in \Omega_{f}}\;{\left(f-\mu \left(\xi_{\text{q}}^{-1}\right)\right)}^{2}d\text{x}=\lambda \int_{\text{x}\in \Omega_{f}}\;\left(\frac{\partial \mu \left(\xi_{\text{q}}^{-1}\right)}{\partial q_{i}}\frac{\partial \mu \left(\xi_{\text{q}}^{-1}\right)}{\partial q_{j}}+\left(\mu \left(\xi_{\text{q}}^{-1}\right)-f\right)\frac{\partial^{2}u\left(\xi_{\text{q}}^{-1}\right)}{\partial q_{i}\partial q_{j}}\right)d\text{x}$$

On average, $\mu \left(\xi_{\text{q}}^{-1}\right)-f$ should be zero at the solution, so the second term can be omitted to obtain a positive definite approximation:

(A7) $$\lambda \int_{\text{x}\in \Omega_{f}}\;\frac{\partial \mu \left(\xi_{\text{q}}^{-1}\right)}{\partial q_{i}}\frac{\partial \mu \left(\xi_{\text{q}}^{-1}\right)}{\partial q_{j}}d\text{x}=\lambda \int_{\text{x}\in \Omega_{f}}\;\left(\frac{\partial \xi_{\text{q}}^{-1}}{\partial q_{i}}\cdot \left(\left(\nabla \mu \right)\circ \xi_{\text{q}}^{-1}\right)\right)\left(\frac{\partial \xi_{\text{q}}^{-1}}{\partial q_{j}}\cdot \left(\left(\nabla \mu \right)\circ \xi_{\text{q}}^{-1}\right)\right)d\text{x}$$

Finally, a change of variables gives:

(A8) $$\lambda \int_{\text{x}\in \Omega^{\prime }}\;\left|\text{D}\xi_{\text{q}}\right|\left(\left(\frac{\partial \xi_{\text{q}}^{-1}}{\partial q_{i}}\circ \xi_{\text{q}}\right)\cdot \left(\nabla \mu \right)\right)\left(\left(\frac{\partial \xi_{\text{q}}^{-1}}{\partial q_{j}}\circ \xi_{\text{q}}\right)\cdot \left(\nabla \mu \right)\right)d\text{x}.$$

### B. Template gradients

The gradient in equation (23) is not the same as the spatial gradient of the template image (*μ*), which would be computed via

(A9) $$\nabla \mu =\frac{\sum_{n=1}^{N}\;\left|\text{D}\phi_{{\text{v}}_{n}}\right|\nabla \left(f_{n}\circ \phi_{{\text{v}}_{n}}\right)+\sum_{n=1}^{N}\;\left(f_{n}\circ \phi_{{\text{v}}_{n}}\right)\nabla \left|\text{D}\phi_{{\text{v}}_{n}}\right|}{\sum_{n=1}^{N}\;\left|\text{D}\phi_{{\text{v}}_{n}}\right|}-\frac{\left(\sum_{n=1}^{N}\;\left|\text{D}\phi_{{\text{v}}_{n}}\right|\left(f_{n}\circ \phi_{{\text{v}}_{n}}\right)\right)\left(\sum_{n=1}^{N}\;\nabla \left|\text{D}\phi_{{\text{v}}_{n}}\right|\right)}{{\left(\sum_{\text{n}=1}^{n}\;\left|\text{D}\phi_{{\text{v}}_{n}}\right|\right)}^{2}}.$$

The above expression incorporates the gradient of the Jacobian determinants, which would have a detrimental effect on the registration (see Appendix C). The Gâteaux variation of the matching term, with respect to variations in the initial velocity, is

(A10) $$\begin{array}{l} \;{\frac{d}{d\tau }\frac{\lambda_{n}}{2}\int_{\text{x}\in \Omega_{\mu }}\left|\text{D}\phi_{{\text{v}}_{n}}\left(\text{x}\right)\right|{\left(f_{n}\circ \phi_{{\text{v}}_{n}}\left(\text{x}\right)-\mu \left(\text{x}-\tau \text{h}\left(\text{x}\right)\right)\right)}^{2}d\text{x}|}_{\tau =0}\\ {=\frac{d}{d\tau }\frac{\lambda_{n}}{2}\int_{\text{x}\in \Omega_{\mu }}\left|\text{D}\phi_{{\text{v}}_{n}}\left(\text{x}\right)\right|{\left(f_{n}\circ \phi_{{\text{v}}_{n}}\left(\text{x}\right)-\frac{\sum_{l=1}^{N}\lambda_{l}\left|\text{D}\phi_{{\text{v}}_{l}}\left(\text{x}\right)\right|f_{l}\circ \phi_{{\text{v}}_{l}}\left(\text{x}-\tau \text{h}\left(\text{x}\right)\right)}{\sum_{l=1}^{N}\lambda_{l}\left|\text{D}\phi_{{\text{v}}_{l}}\left(\text{x}\right)\right|}\right)}^{2}d\text{x}|}_{\tau =0}\\ =\lambda_{n}\int_{\text{x}\in \Omega_{\mu }}\left|\text{D}\phi_{{\text{v}}_{n}}\left(\text{x}\right)\right|\left(f_{n}\circ \phi_{{\text{v}}_{n}}\left(\text{x}\right)-\mu \left(\text{x}\right)\right)g\left(\text{x}\right)\cdot \text{h}\left(\text{x}\right)d\text{x} \end{array}$$

where *μ* and **g** are computed as in equations (21) and (23). A similar scheme may be used to derive the positive definite approximation to the second Gâteaux variation.

For the software implementation, the gradients of the warped images are computed by sampling the image and its gradients according to the transformation, and multiplying the gradients by the transpose of the Jacobian tensor at each point.

(A11) $$\nabla \left(f_{n}\circ \phi_{{\text{v}}_{n}}\right)={\left(\text{D}\phi_{{\text{v}}_{n}}\right)}^{T}\left(\left(\nabla f_{n}\right)\circ \phi_{{\text{v}}_{n}}\right)$$

### C. Pairwise symmetry

Pairwise symmetric registration is a special case of the group-wise formulation, and is of interest to many. We note that for pairwise registration (i.e., *N* = 2), where we define **v** = **v**_1_ = −**v**_2_ (and the regularization term has been halved for convenience), the objective function reduces to

(A12) $$\varepsilon =½\int_{\text{x}\in \Omega_{\mu }}\frac{\lambda_{1}\lambda_{2}\left|\text{D}\phi_{\text{v}}(\text{x})\right|\left|\text{D}\phi_{-\text{v}}(\text{x})\right|}{\lambda_{1}\left|\text{D}\phi_{\text{v}}(\text{x})\right|+\lambda_{2}\left|\text{D}\phi_{-\text{v}}(\text{x})\right|}{\left(f_{1}\circ \phi_{\text{v}}(\text{x})-f_{2}\circ \phi_{-\text{v}}(\text{x})\right)}^{2}d\text{x+}½{\left\|{\text{L}}_{\text{v}}\text{v}\right\|}^{2}$$

The solution, where derivatives of the objective function with respect to variations in **v** are zero, satisfies the following:

(A13) $$\frac{\lambda_{1}\lambda_{2}\left|\text{D}\phi_{\text{v}}\right|\left|\text{D}\phi_{-\text{v}}\right|(\lambda_{1}\left|\text{D}\phi_{\text{v}}\right|\nabla (f_{1}\circ \phi_{\text{v}})+\lambda_{2}\left|\text{D}\phi_{-\text{v}}\right|\nabla (f_{2}\circ \phi_{-}))\;(f_{2}\circ \phi_{-\text{v}}-f_{1}\circ \phi_{\text{v}})}{{\left(\lambda_{1}\left|\text{D}\phi_{\text{v}}\right|+\lambda_{2}\left|\text{D}\phi_{-\text{v}}\right|\right)}^{2}}={\text{L}}_{\text{v}}^{†}{\text{L}}_{\text{v}}\text{v}.$$

For the geodesic shooting in approach to work, we need to consider the solution along the entire trajectory of the diffeomorphism. To do this, we introduce another diffeomorphic mapping, **ζ**, which is used to assess the effect of determining the initial velocities at some point other than the mid-point. For the geodesic shooting in equation (19) to work properly, the left-hand side of equation (45) (which we here refer to as momentum, **u**) must become |**D*****ζ***(**D*****ζ***)*^T^*(**u** ∘ **ζ**)| if we replace (**ϕ_v_** and ***ϕ***_−**v**_ with (**ϕ_v_** ∘ **ζ** and **ϕ**_−**v**_ ∘ **ζ** respectively. For typesetting reasons, we decompose the left-hand side of equation (45) into two factors and consider what happens to each of them if we make those substitutions.

For the first factor [corresponding to *a_n_*(**x**) in equation 22], using the identity |**D**(**ϕ** ∘ **ζ**)| = |**Dζ**|(|**Dϕ**| ∘ **ζ**) we obtain

(A14) $$\frac{\lambda_{1}\lambda_{2}\left|\text{D}(\phi_{\text{v}}\circ \zeta )\right|\left|\text{D}(\phi_{-\text{v}}\circ \zeta )\right|(f_{1}\circ \phi_{\text{v}}\circ \zeta -f_{2}\circ \phi_{-\text{v}}\circ \zeta )}{\lambda_{1}|\text{D}(\phi_{\text{v}}\circ \zeta )|+\lambda_{2}|\text{D}(\phi_{-\text{v}}\circ \zeta )|}=|\text{D}\zeta |\left(\frac{\lambda_{1}\lambda_{2}\left|\text{D}\phi_{\text{v}}\right|\left|\text{D}\phi_{-\text{v}}\right|(f_{1}\circ \phi_{\text{v}}-f_{2}\circ \phi_{-\text{v}})}{\lambda_{1}|\text{D}\phi_{\text{v}}|+\lambda_{2}|\text{D}\phi_{-\text{v}})|}\right)\circ \zeta \text{.}$$

For the second factor [corresponding to **g**(**x**) in equation 22], we in addition use the element-wise matrix multiplication $\nabla \left(f\circ \zeta \right)={\left(\text{D}\zeta \right)}^{T}\left(\nabla f\right)\circ \zeta$ to obtain

(A15) $$\frac{\lambda_{1}\left|\text{D}(\phi_{\text{v}}\circ \zeta )\right|\nabla (f_{1}\circ \phi_{\text{v}}\circ \zeta )+\lambda_{2}\left|\text{D}(\phi_{-\text{v}}\circ \zeta )\right|\nabla (f_{2}\circ \phi_{-\text{v}}\circ \zeta )}{\lambda_{1}|\text{D}(\phi_{\text{v}}\circ \zeta )|+\lambda_{2}|\text{D}(\phi_{-\text{v}}\circ \zeta )|}={\left(\text{D}\zeta \right)}^{T}\left(\frac{\lambda_{1}\left|\text{D}\phi_{\text{v}}\right|\nabla (f_{1}\circ \phi_{\text{v}})+\lambda_{2}\left|\text{D}\phi_{-\text{v}}\right|\nabla (f_{2}\circ \phi_{-\text{v}})}{\lambda_{1}|\text{D}\phi_{\text{v}}|+\lambda_{2}|\text{D}\phi_{-\text{v}})|}\right)\circ \zeta \text{.}$$

By recombining the factors, we see that symmetry is achieved, while still satisfying the requirements of a geodesic shooting approach based on the *EPdiff* equation (Euler-Poincaré equation on diffeomorphisms).

This symmetric registration approach is very similar to that of Hart et al. (2009), Niethammer et al. (2009), although others have proposed different strategies for achieving inverse consistency. Previously Beg and Khan (2007) presented two adjustments to enforce inverse consistency in the large deformation diffeomorphic metric mapping (LDDMM) approach. The first of these was based on aligning an image pair to their simple half-way average. It is similar to the approach of Avants et al. (2008), as well as one of the approaches proposed in Younes (2007). Unfortunately, it introduces a discontinuity in the evolution equations at the mid-point (Bruveris et al., 2011). The second approach of Beg and Khan (2007) does not introduce this discontinuity, but leads to more complicated evolution equations that do not strictly obey *EPdiff*. Similarly, the other strategy for achieving inverse consistency proposed in Younes (2007) also does not obey these equations.

We note that our matching term in equation (A12) does not satisfy all the desiderata set out by Tagare et al. (2009). In particular, we see that for aligning two constant images, this term will depend on the deformations. However, it is worth noting that the derivatives of the matching term with respect to velocity (see equation A13) are zero for constant images, so there should not be any tendency toward favoring any particular solution.

## Footnotes

^1^Following Tagare et al. (2009) we prefer the term “symmetric” to alternatives such as “inverse-consistent” because of its formal mathematical definition, which includes not only invariance to swapping a pair of images, but also invariance to permutations of more than two images.

^2^See also the FSL SIENA tool, www.fmrib.ox.ac.uk/fsl/siena/index.html

^3^Available from www.fil.ion.ucl.ac.uk/spm/

^4^See http://nifti.nimh.nih.gov/nifti-1

^5^Bi-invariance requires that composing both transformations with a third should not change their distance, and that inverting both should also preserve the distance; in the simple positive scalar case, the logarithmic distance between 1/2 and 1/4 is the same as that between 1/6 and 1/12, and the same as that between 2 and 4.

^6^Freely available from http://www.oasis-brains.org/

^7^We note that this is the approach typically adopted within the SPM software, and that parallel transport (Younes et al., 2008) should instead have been used to warp the velocity fields to MNI space, prior to computing their divergences.

^8^The alternative is to use small deformation assumptions, which suppose that deviations from the identity transform can constructed by addition of displacements, rather than composing a series of tiny deformations.
